# Surveillance for *Trichinella* infection in U.S. pigs raised under controlled management documents negligible risk for public health

**DOI:** 10.1016/j.fawpar.2024.e00238

**Published:** 2024-07-22

**Authors:** H. Ray Gamble, Dolores E. Hill, Valsin Fournet, Brandon Adams, Diane Hawkins-Cooper, Jorrell Fredericks, Jovan Aquino, Sonia Agu, Nadya Chehab, Ako Ankrah, Maria C. Antognoli, Marta D. Remmenga, Scott Kramer, Lori Gustafson, Benjamin M. Rosenthal

**Affiliations:** a U.S. Department of Agriculture, Animal Parasitic Disease Laboratory, Beltsville, MD 20705, USA; b U.S. Department of Agriculture, Animal and Plant Health Inspection Service, Veterinary Services, Fort Collins, CO 80526, USA; c U.S. Department of Agriculture, Animal and Plant Health Inspection Service, Veterinary Services, Riverdale, MD 20737, USA

**Keywords:** Trichinella, Surveillance, Negligible risk, Controlled management

## Abstract

Biosecurity measures preventing exposure of pigs to rodents, wildlife, and contaminated feed or waste products reduce the risk of zoonotic *Trichinella* infection in pork. To understand the benefits of such measures in the United States, we conducted the first comprehensive survey of pigs produced under the Pork Quality Assurance Plus production standard, surveying 3,208,643 pork samples from twelve processing locations tested over a period of 54 months. We detected no *Trichinella* sp. positives in any of these pork samples, providing a 95% confidence in a *Trichinella* sp. prevalence of <1 in 1,000,000 for the processors represented by the study. These results are consistent with international guidelines for having a negligible risk to public health. Results obtained here should generalize to all PQA+ sources, as *Trichinella* sp. exposure risk is based on production guidelines that extend to the larger PQA+ population.

## 1 Introduction

The occurrence of *Trichinella* sp. in pork once posed a major food safety risk in many countries, justifying decades of effort expended on controlling this parasite. With the advent of modern pork production practices, beginning with reforms in waste feeding and continuing with the introduction of modern bio-secure management systems, *Trichinella* sp. is no longer present in commercial pork production where these practices have been adopted and maintained (Gamble, 2022). Risk does remain where pigs are raised under uncontrolled conditions, such as free-range management which allows contact with wildlife, or where hygienic standards facilitate rodent infestation or include feeding of animal carcasses or uncooked meat waste (Gamble, 2022).

Historically, prevention of human exposure to *Trichinella* sp. in pork has been achieved by individual carcass testing at slaughter, and by post-slaughter treatments such as freezing, heating, or certain methods of curing (Noeckler et al., 2019). National and international regulations governing intra- and interstate commerce specify these protective measures (European Community, 2015; World Organisation for Animal Health, 2023a), and adherence to these regulations often impacts international trade agreements. The mitigation of risk of exposure to *Trichinella* sp. achieved by modern pork production systems renders superfluous costly post-harvest safeguards such as individual carcass testing, excessive heating, freezing, and other processing methods. In particular, the considerable cost of carcass testing is redundant for populations of pigs where *Trichinella* sp. infection no longer occurs (Pozio, 1998).

Modern pork production systems incorporate biosecurity measures that protect animal and human health. These production measures, including confinement housing, food security and facility hygiene, evolved initially to prevent devastating viral diseases such as hog cholera. A subset of controlled management parameters has been defined by pork producers and regulatory authorities for raising pigs under conditions of controlled management that eliminate exposure of pigs to sources of *Trichinella* sp.; these biosecurity standards exclude wildlife, rodents, and contaminated feed from contact with pigs (Gamble et al., 2019). The conditions for controlled management are articulated in guidelines of the World Organisation for Animal Health (WOAH) (World Organisation for Animal Health, 2023a) and are referenced in national pork product standards. In pork production systems adhering to controlled management, *Trichinella* sp. infection is absent based on results of routine slaughter testing (Gamble, 2022).

In the United States, U.S. Pork Quality Assurance Plus (PQA+) (https://porkcheckoff.org/certification-tools/training-certification/pqa-plus/) is an education and certification program designed to help pig farmers and their employees continually improve production practices. It addresses food safety, animal well-being, environmental stewardship, worker safety, and public health. Specific for prevention of exposure to *Trichinella* sp.*,* PQA+ includes five guidelines outlining best management practices:

• Following proper feed biosecurity protocols.
• Preventing exposure to rodents, wildlife, and birds.
• Refraining from feeding raw food waste of animal origin to swine.
• Promptly removing and properly disposing of swine carcasses.
• Documenting animal arrivals and departures from PQA+ production sites.

PQA+ guidelines, and the details for implementation, follow those for controlled management described by WOAH (World Organisation for Animal Health, 2023a) and guidelines of the International Commission on Trichinellosis (Gamble et al., 2019). PQA+ is compliant with International Organisation for Standardization (ISO) Technical Specification (TS) 34700; compliance is assured by auditing. There are currently >63,000 U.S. pork producers certified under PQA+ (U.S. National Pork Board, pers. comm.).

International standards consider controlled management programs, such as PQA+, suitable for assuring “negligible risk” for *Trichinella* sp. (Codex Alimentarius, 2015). Codex Alimentarius (Codex) guidance states that compliance auditing or surveillance can provide assurance once a population of pigs has been defined as raised under controlled management. Specifically, for surveillance, testing data should demonstrate that “prevalence of infection does not exceed 1 infected carcass per 1,000,000 pigs slaughtered with at least 95% confidence.” Various studies in the U.S. have demonstrated low prevalence of absence of infection with *Trichinella* sp. in domestic pigs (Davies et al., 1998; Gamble and Bush, 1998). However, these studies were not focused on a defined population of pigs raised under controlled management (PQA+), did not include a broad distribution of samples from all major pig producing states, and did not test to an internationally accepted target prevalence. The objective of the current study was to surveil U.S. pigs raised under PQA+ across a distributed sample of pig-productions sites, for prevalence of *Trichinella* sp. infection using the statistical parameters for negligible risk of <1/1 M infections as established by the Codex Alimentarius.

## 2 Methods

### 2.1 Sampling design

**Fig. 1:**
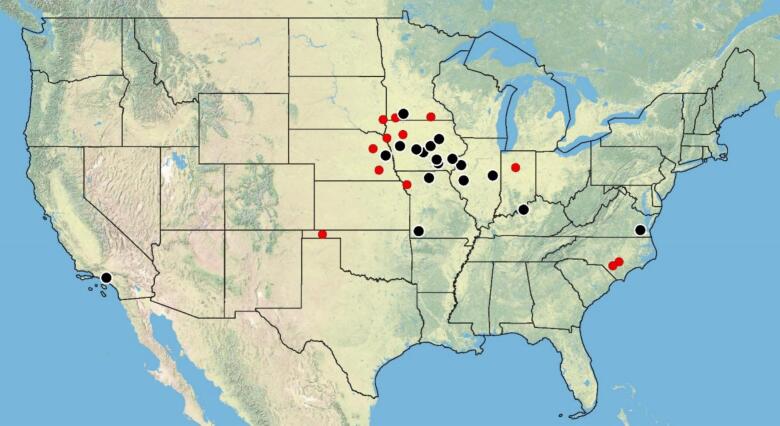
Map of 30 pork processing plants accounting for slaughter of >95% of PQA Plus pigs in the United States. Locations submitting samples are designated in red. (For interpretation of the references to colour in this figure legend, the reader is referred to the web version of this article.)

A total of 11 U.S. pork processing companies, including 30 locations in 12 states, account for >95% of all PQA+ market pigs in the U.S (National Pork Board, pers. comm.) (Fig. 1). At the beginning of this study, the daily count of PQA+ pigs processed at these 30 locations was 456,200. Based on these numbers, and approximating the annual cycle, an initial sample size of 3.1 million (M), collected over a period of 60 weeks, was selected to fit a design prevalence demonstrating infection of <1 positive/1 M pigs (Cannon, 2001). This calculation assumed diagnostic specificity and sensitivity of 100 and 95%, respectively, required for the testing protocol to confirm a positive case. After the global pandemic forced facility closure and testing delays, we increased the sample size to 3.138 M to meet the same design prevalence for the larger population of PQA+ pigs harvested during that extended interval. The extension to a longer timeframe was considered justifiable as the expected prevalence of *Trichinella* sp., and the potential for new exposure, was negligible or stable throughout the study period.

For the purposes of this study, all facilities processing PQA+ pigs were considered similar in *Trichinella* sp. risk based on adherence of PQA+ producers to protective biosecurity standards. From the 30 locations processing PQA+ pigs, 12 processing locations in 8 states agreed to participate and provided samples from most states (23 in total, see Table 1) that participate in the PQA+ program. Samples requested from each location were determined based on the relative percentage of pigs processed by the company. The number of weekly samples initially requested by processing plant location ranged from 2121 to 12,735.

**Table 1:** Samples by state of origin of pigs tested.

**STATE**	**SAMPLES TESTED**	**% TOTAL TESTED**
Arkansas	73	0.002
Colorado	35,875	1.118
Georgia	931	0.029
Indiana	202,885	6.323
Illinois	23,074	0.719
Iowa	917,785	28.604
Kansas	107,315	3.345
Kentucky	1083	0.034
Michigan	4376	0.136
Minnesota	824,520	25.697
Missouri	5349	0.167
Nebraska	176,657	5.506
North Carolina	552,004	17.204
North Dakota	3637	0.113
Ohio	30,253	0.943
Oklahoma	62,823	1.958
Pennsylvania	2266	0.071
South Carolina	15,398	0.480
South Dakota	146,319	4.560
Tennessee	1363	0.042
Texas	36,947	1.151
Virgina	57,130	1.781
Wisconsin	580	0.018
**TOTAL**	3,208,643	100

### 2.2 Sample collection

Sample bags, labels, and shipping materials were provided by the USDA to participating pork processing facilities. Samples of approximately five grams (g) were collected from the tip of the pillars of the diaphragm (crus muscle). A total of 100 samples, collected at random, were placed in each bag. Bags were numbered and labeled with the date of collection. Details of collected samples, including the state or premise of origin, were entered into spreadsheets, and sent to the testing laboratory.

### 2.3 Testing methods

All samples were submitted to the U.S. Department of Agriculture, Animal Parasitic Diseases Laboratory in Beltsville, Maryland USA. Upon receipt, samples were logged for location and date of sample collection. Prior to testing, samples were retained under refrigeration (4 °C).

Testing for *Trichinella* sp. infection was performed using a standard pooled sample digestion method as described extensively elsewhere (Gajadhar et al., 2019; World Organisation for Animal Health, 2023b; International Organisation for Standards (ISO), 2015). Samples were cut and weighed to ∼1 g (but no >1.5 g) and the remaining tissue was retained pending the outcome of test results. Pools of 100 samples were ground in a blender, then added to a 3-l (L) beaker containing 2 L of water with 10.8 mL of 12 N hydrochloric acid (HCl) and ten grams of pepsin. The digestion mixture was covered with aluminum foil, a magnetic bar added and stirred on stir plate in an incubator at a constant temperature of 43–45 °C for one hour. The digestion mixture was then removed, checked for completion of digestion, then poured through a 180 μm mesh sieve into a 2-l separatory funnel. An additional 100 mL of tap water was used to rinse the beaker and the sieve into the 2-l separatory funnel. After a 30-min sedimentation period, 40 mL of the digestion fluid was drained from the separatory funnel into a 50 mL centrifuge tube and settled for 10 min. After initial settling, all but 20 mL was poured off, retaining the sediment. Tap water was then added to a final volume of 40–50 mL, and this was allowed to settle for another 10 min. This procedure was repeated (usually 2 or 3 times) until the sample cleared of host tissue debris. The final sediment was poured into a gridded Petri dish and examined under a stereomicroscope at 20× for the presence of *Trichinella* sp. larvae. Results were recorded in spreadsheets along with date of sample collection, state of origin and test result. Sufficient metadata were retained to ensure the possibility of traceback in the event that one or more samples tested positive.

The proficiency of each person performing testing was assured by means of thorough training and quarterly assessment of their ability to distinguish infected from uninfected test samples and enumerate, within acceptable bounds of error, the number of larvae placed in test samples prepared for this purpose. Proficiency testing was performed according to methods used in the USDA, Agricultural Marketing Service, Trichinae Export Program Laboratory Manual, and conforming to the standards included in ISO/IEC 17025:2017- General requirements for the competence of testing and calibration laboratories and recommendations of the International Commission on Trichinellosis (Gajadhar et al., 2019). Following initial training, analysts were subjected to quarterly testing including four unknown samples (two positive and two negative samples). Successful performance of proficiency testing required all samples to be correctly identified. Failure of proficiency testing excluded analysts from examining samples until completing further training. More than fifty such technicians (many of whom we gratefully thank in the acknowledgement section) contributed to completing this ambitious project, performing up to 100 digestions on any given day (obeying changing demands of social distancing through and following the COVID-19 public health emergency).

## 3 Results

Sampling began in August 2019 with the initial goal of completing testing by the end of 2020. From August 2019 through March 2020, sample testing averaged approximately 52,000/week. The outbreak of Covid-19 forced suspension of testing in March 2020; a smaller team commenced socially-distanced testing in July 2021 and continued, at an increasing pace from 10,000 - 20,000/week, through December 2023.

A total of 3,208,643 samples from PQA+ pigs were tested by the pooled sample digestion method. Among samples collected, 77.6% included information on the state of origin and/or producer name. For samples lacking these data, state of origin was extrapolated based on the pro rata distribution of state of origin for each packer that submitted samples. In total, samples were obtained from pigs produced in 23 different states. A breakdown of these samples by state of origin is shown in Table 1. *Trichinella* sp. was not detected in any of the 3,208,643 tested samples.

## 4 Discussion

International guidelines consider a *Trichinella* sp. infection prevalence of <1 in 1,000,000 pigs to be a prerequisite for defining a population of pigs as having a negligible risk to human health (World Organisation for Animal Health, 2023a; Codex Alimentarius, 2015). In this study we detected no *Trichinella* sp. positive pigs in over 3.2 million samples collected over a 54-month period from pigs enrolled in the U.S. PQA+ program and originating from 23 U.S. states. These data affirm prior studies establishing that biosecurity measures, wherever consistently applied, render as negligible the risk posed by *Trichinella* sp.to pork (i.e. Gottstein et al., 2009; Alban and Petersen, 2016; Franssen et al., 2018; Gamble et al., 2019; Pozio et al., 2010; Alban et al., 2008; Gamble, 2022).

Sampling locations were a voluntary subset of U.S. packers that process pigs from PQA+ facilities and these data document that *Trichinella* sp. prevalence is <1 in 1,000,000 pigs (at a 95% confidence level) across the study locations and time period. It is important to note that negative test results are only indicative of absence of infection at the level of test sensitivity. Prior data establish the sensitivity of the test used here as approximately 3 larvae per gram of tissue (Gamble, 1998). It is conceivable that low levels infections were not detected. Nevertheless, our testing methods are considered the gold standard method as referenced by the World Organisation for Animal Health and national authorities (European Community, 2015). Thus, the results reported here, demonstrating absence of infection are consistent with all other testing results using these standard methods.

Generalization of conclusions throughout the full PQA+ population is justified because all processors of PQA+ pigs, at the point of sale, document producer adherence to common biosecurity measures. We found no evidence of infection in pigs sampled from any participating plant. Each plant sampled broadly and blindly from PQA+ producers; producers were unaware of testing being performed and therefore could not knowingly bias the sample with respect to *Trichinella* sp. risk. Although underlying risk may vary among producers, we neither documented nor had reason to suspect that risk varied systematically among the PQA+ producers. The risk of *Trichinella* sp. exposure in PQA+ pigs processed at unsampled facilities was likely no greater than risk in pigs processed at sampled facilities. Therefore, these data fairly reflect PQA+ producers as a whole and demonstrate success in mitigating risk.

This study is the most comprehensive and ambitious survey for *Trichinella* sp. in pigs ever conducted in the United States, and the results accord with data derived from other surveys documenting how controlled management systems for pork production have reduced the occurrence of *Trichinella* sp. in U.S. pork (Gamble, 2006; Gamble, 2022). For example, during the period of 1996 to 2016, a U.S. Department of Agriculture voluntary export testing program reported a total of 38,755,374 samples tested, all of which were negative for *Trichinella* sp. infection (USDA, Agricultural Marketing Service, pers. comm.). The absence of public health reports of pork-derived human trichinellosis cases further substantiates the absence of infection in U.S. commercial pork (Centers for Disease Control and Prevention, 2017).

Historically, processed (including ready-to-eat) products were governed by specific rules for heating, freezing, or curing. In the U.S., rules formerly found in the U.S. Code of Federal Regulations (9CFR 318.10), have been rescinded and replaced by compliance guidance under which pork processors are required to assess the likelihood that *Trichinella* sp. poses a public health risk (FSIS, 2018). The data reported here support the expectation that pork from pigs managed under the PQA+ program do not pose a public health risk, thereby not requiring further testing or processing as a mitigation for risk from *Trichinella* sp.

## 5 Conclusions

The surveillance data reported here support the assertion that the U.S. PQA+ program is an effective mitigation of risk for *Trichinella* sp. infection in pigs. Demonstrating that the prevalence of *Trichinella* sp. infection is <1 in 1 million pigs in facilities processing PQA+ pigs may allow for further consideration of this population as having a negligible risk. Further, the data reported here could be useful for HACCP decisions where labelling or further processing requires assessment of risk from *Trichinella* sp.

## CRediT authorship contribution statement

**H. Ray Gamble:** Writing – review & editing, Writing – original draft, Project administration, Investigation, Funding acquisition, Data curation. **Dolores E. Hill:** Writing – review & editing, Project administration, Methodology, Investigation, Conceptualization. **Valsin Fournet:** Writing – review & editing, Project administration, Methodology, Investigation, Data curation. **Brandon Adams:** Writing – review & editing, Investigation, Data curation. **Diane Hawkins-Cooper:** Writing – review & editing, Project administration, Methodology, Investigation, Conceptualization. **Jorrell Fredericks:** Writing – review & editing, Investigation. **Jovan Aquino:** Writing – review & editing, Methodology, Investigation. **Sonia Agu:** Writing – review & editing, Methodology, Investigation. **Nadya Chehab:** Writing – review & editing, Project administration, Investigation. **Ako Ankrah:** Writing – review & editing, Methodology, Investigation. **Maria C. Antognoli:** Writing – review & editing, Methodology, Data curation, Conceptualization. **Marta D. Remmenga:** Writing – original draft, Methodology, Formal analysis, Data curation, Conceptualization. **Scott Kramer:** Writing – review & editing, Methodology, Formal analysis. **Lori Gustafson:** Writing – review & editing, Formal analysis, Conceptualization. **Benjamin M. Rosenthal:** Writing – review & editing, Writing – original draft, Visualization, Validation, Supervision, Project administration, Methodology, Investigation, Funding acquisition, Formal analysis, Conceptualization.

## Declaration of competing interest

The authors declare the following financial interests/personal relationships which may be considered as potential competing interests:

Benjamin Rosenthal reports financial support was provided by National Pork Producers Council. H. Ray Gamble reports a relationship with National Pork Producers Council that includes: consulting or advisory. H. Ray Gamble is on the Editorial Board of Food and Waterborne Parasitology. B.M. Rosenthal has been invited to consider serving on the Editorial Board of Food and Waterborne Parasitology. If there are other authors, they declare that they have no known competing financial interests or personal relationships that could have appeared to influence the work reported in this paper
